# Physiological and Transcriptomic Characterization of Salt-Stress Responses in the Pyrethrin-Producing Ornamental Plant *Tanacetum coccineum*

**DOI:** 10.3390/plants15213496

**Published:** 2026-11-09

**Authors:** Daju Chen, Wenqing Zhang, Tuo Zeng, Caiyun Wang

**Affiliations:** 1 Guizhou Key Laboratory of Forest Cultivation in Plateau Mountain, College of Life Sciences, Guizhou Normal University, Guiyang 550025, China; cdj8@gznu.edu.cn (D.C.); zhangwq@gznu.edu.cn (W.Z.); 2 National Key Laboratory for Germplasm Innovation & Utilization of Horticultural Crops, College of Horticulture & Forestry Sciences, Huazhong Agricultural University, Wuhan 430070, China; wangcy@mail.hzau.edu.cn

**Keywords:** salinity, WGCNA, antioxidant defense, ABA biosynthesis, pyrethrins

## Abstract

*Tanacetum coccineum* is an ornamental plant capable of producing pyrethrins, natural insecticidal metabolites. Salinity restricts plant growth, but its effects on stress responses and pyrethrin-related metabolism remain poorly understood in this species. In this study, seedlings were treated with 0, 100, or 200 mM NaCl for 7 days, followed by physiological measurements and transcriptome analysis of leaf and root. Salt stress caused progressively more severe leaf injury and distinct antioxidant responses in the two tissues. Transcriptome analysis revealed extensive tissue-dependent transcriptional changes, including differential expression of genes associated with antioxidant defense, ABA biosynthesis, and pyrethrin metabolism. Several pyrethrin biosynthesis-related genes were upregulated under moderate salinity, whereas their expression patterns became more variable at higher NaCl concentrations. Meanwhile, spectrophotometrically estimated leaf pyrethrin content increased with NaCl concentration. WGCNA and PPI network analyses identified candidate hub genes associated with ion homeostasis, oxidative defense and carbon metabolism. Further analysis identified 19 candidate transcription factors and significant co-expression associations with ABA- and pyrethrin-related genes, suggesting potential transcriptional links between salt-stress responses and specialized metabolism. Collectively, these findings reveal tissue-dependent responses to salinity and provide insights into the transcriptional complexity of pyrethrin-related metabolism in *T. coccineum*.

## 1. Introduction

*Tanacetum coccineum* (syn. *Chrysanthemum coccineum*), also known as Persian pyrethrum, is an herbaceous perennial in the Asteraceae family. It is widely cultivated as an ornamental for its large, vibrant pink to red flower heads and as a source of natural pyrethrins with insecticidal properties [1]. The species has a long history of use as a botanical insecticide, with pyrethrin-related applications dating back to ancient Persia as early as 400 BC [2]. However, its current utilization differs substantially from that of the closely related *T. cinerariifolium*. Because *T. cinerariifolium* generally accumulates considerably higher levels of pyrethrins, it has become the principal species used for modern commercial pyrethrin production, whereas *T. coccineum* is primarily valued as an ornamental plant [3]. Previous studies of *T. coccineum* have mainly focused on the tissue-specific distribution and biosynthesis of pyrethrins during floral development [4]. In contrast, relatively little is known about its physiological and transcriptional responses to adverse environmental conditions, particularly salinity, or whether salinity affects pyrethrin metabolism.

Soil salinity is an important environmental constraint that can impair plant establishment, vegetative growth, and ornamental quality through osmotic stress, ionic imbalance, and oxidative damage [5,6]. Salt stress drives reactive oxygen species (ROS) accumulation, which can lead to membrane lipid peroxidation, while antioxidant enzymes such as catalase (CAT), peroxidase (POD), and superoxide dismutase (SOD) contribute to maintaining cellular redox homeostasis [7,8]. These physiological responses are often accompanied by extensive transcriptional and metabolic adjustments. In addition to affecting primary metabolism, salinity can alter secondary metabolism, although the direction and magnitude of these changes vary among species, tissues, metabolites, and stress intensities [9,10]. Among the hormonal pathways involved in these responses, abscisic acid (ABA) is an important regulator linking salt-stress signaling with transcriptional and metabolic adjustments. ABA-associated signaling has been linked to changes in flavonoid and phenylpropanoid metabolism in species such as *Glycyrrhiza uralensis* and rice [11,12]. Transcriptomic analysis of *Melilotus albus* further identified ABA-responsive gene modules associated with secondary metabolism, including a module containing the hub gene *4CL1*, suggesting a potential connection between ABA-mediated stress responses and secondary metabolism [13]. Salt stress and ABA can also alter the expression of genes involved in carotenoid and ABA biosynthesis, including *phytoene synthase* and *9-cis-epoxycarotenoid dioxygenase* (*NCED*), thereby contributing to ABA-associated stress responses and metabolic adjustments [14,15]. Collectively, these studies suggest that antioxidant defense, ABA-associated signaling, and secondary metabolism are closely interconnected components of plant responses to salinity.

Pyrethrin biosynthesis and its transcriptional regulation have been studied more extensively in the commercial pyrethrum species *T. cinerariifolium*. Several transcription factors involved in hormonal regulation of pyrethrin biosynthesis have been functionally characterized. For instance, *TcbZIP60* directly binds to the promoters of *TcCHS* and *TcAOC* and activates their expression in response to methyl jasmonate (MeJA) and ABA [16], whereas *TcWRKY75* positively regulates key genes involved in pyrethrin biosynthesis [17]. *TcMYC2* and *TcMYB8* have also been implicated in jasmonic acid (JA)-mediated regulation of pyrethrin production [18,19]. These studies demonstrate that pyrethrin biosynthesis is subject to complex transcriptional and hormonal regulation. More recently, physiological and transcriptomic analyses of *T. cinerariifolium* showed that drought stress induced pronounced antioxidant and ABA-related transcriptional responses and was accompanied by substantial changes in pyrethrin biosynthesis [20], indicating that pyrethrin metabolism can also respond to abiotic stress. However, despite these advances in *T. cinerariifolium*, studies on *T. coccineum* remain limited. In particular, it remains unclear how *T. coccineum* responds physiologically and transcriptionally to salinity, whether leaves and roots exhibit distinct responses, and whether salt stress affects pyrethrin metabolism.

In this study, *T. coccineum* seedlings were exposed to different NaCl concentrations, and physiological responses were examined separately in leaves and roots to characterize tissue-specific oxidative stress and antioxidant responses. Transcriptome profiling was integrated with weighted gene co-expression network analysis (WGCNA) and predicted protein–protein interaction (PPI) network analysis to identify salt-responsive co-expression modules and candidate hub genes associated with salt stress responses. Additionally, transcript levels of ABA- and pyrethrin-related genes and leaf pyrethrin contents were measured to evaluate the effect of salinity on pyrethrin metabolism. Specifically, this study aimed to address three questions: whether salinity induces distinct physiological responses in leaves and roots of *T. coccineum*; which transcriptional modules and candidate genes are associated with these responses; and whether salt stress alters pyrethrin-related gene expression and pyrethrin accumulation. The results provide a tissue-resolved characterization of salinity responses in *T. coccineum* and identify candidate genes potentially linked to salt-stress response and stress-responsive pyrethrin metabolism.

## 2. Results

### 2.1. Phenotypic and Physiological Responses of T. coccineum to Salt Stress

**Figure 1:**
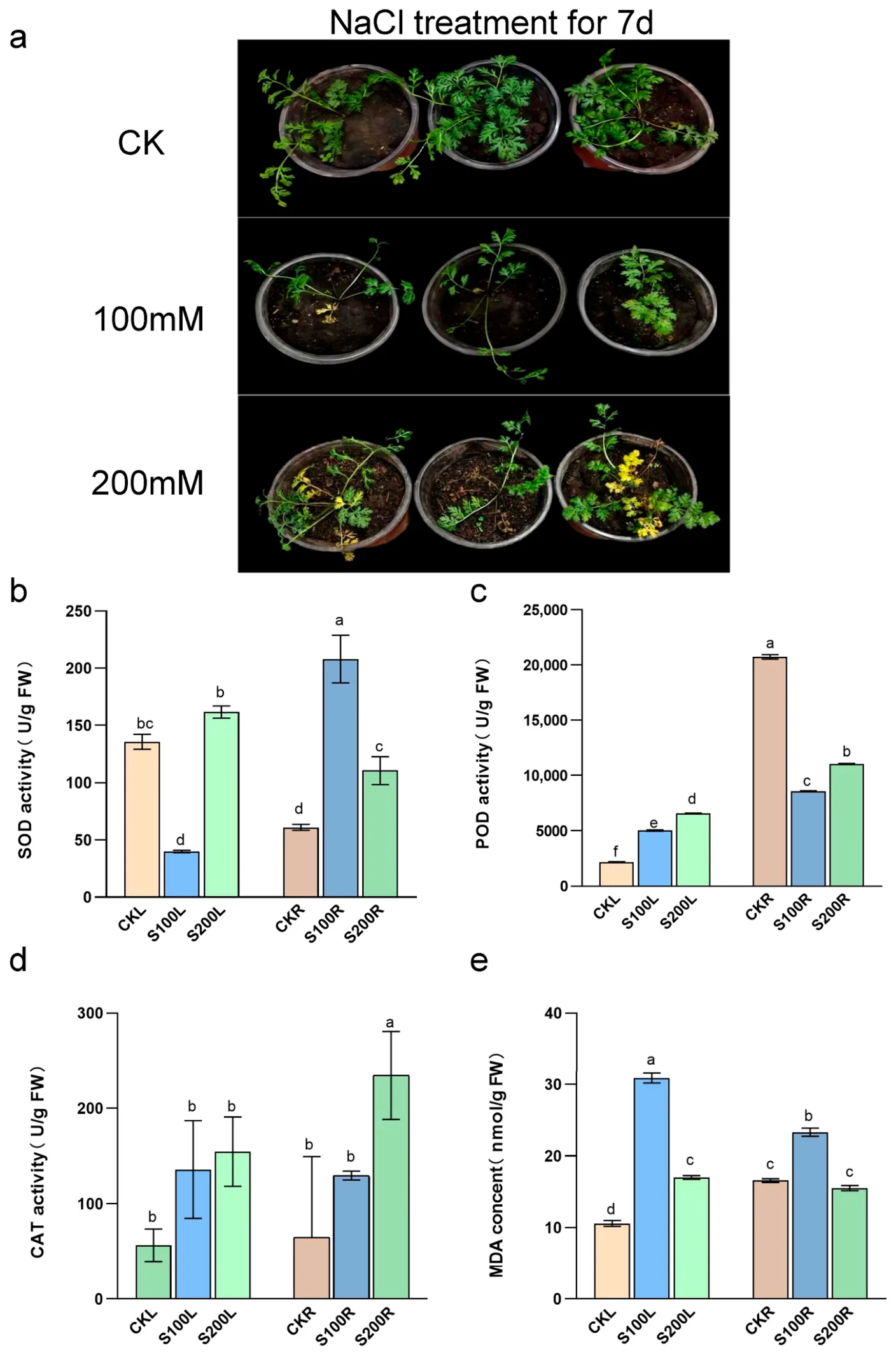
Phenotypic and physiological responses of *T. coccineum* to NaCl stress. (**a**) Representative photographs of plants after 7 days of treatment with 0 (CK), 100, and 200 mM NaCl. (**b**–**e**) Antioxidant enzyme activities and MDA content: (**b**) SOD activity; (**c**) POD activity; (**d**) CAT activity; (**e**) MDA content. CKL, S100L, and S200L represent leaf samples treated with 0, 100, and 200 mM NaCl, respectively; CKR, S100R, and S200R represent the corresponding root samples (L = leaf, R = root). Data are shown as means ± SD. Different lowercase letters indicate significant differences among all six experimental groups, as determined by one-way ANOVA followed by Tukey’s HSD post hoc test (*p* < 0.05).

After 7 days of NaCl treatment, *T*. *coccineum* seedlings exhibited progressively more severe visible injury with increasing NaCl concentration. Plants exposed to 100 mM NaCl showed chlorosis on approximately 1–2 leaves, whereas those treated with 200 mM NaCl developed chlorosis on 3–4 leaves, accompanied by necrosis on 1–2 leaves. In contrast, control plants maintained green foliage without visible chlorosis or necrosis (Figure 1a).

To characterize physiological responses to salinity, POD, CAT, and SOD activities and MDA content were measured separately in leaves and roots (Figure 1b–e). In leaves, SOD activity decreased significantly at 100 mM NaCl and increased again at 200 mM NaCl (Figure 1b). In contrast, POD and CAT activities increased with increasing NaCl concentration (Figure 1c,d). Leaf MDA content increased at 100 mM NaCl and subsequently decreased at 200 mM NaCl, although it remained higher than that of the control (Figure 1e).

Roots exhibited different response patterns. Root SOD activity increased at 100 mM NaCl and then decreased at 200 mM NaCl (Figure 1b), whereas POD activity decreased at 100 mM NaCl and increased again at 200 mM NaCl but remained lower than that of the control (Figure 1c). CAT activity increased with increasing NaCl concentration (Figure 1d). Root MDA content increased at 100 mM NaCl and subsequently decreased at 200 mM NaCl (Figure 1e). Taken together, these results demonstrate that the antioxidant enzyme activities and MDA accumulation responded differently to salinity in leaves and roots.

### 2.2. Transcriptome Assembly, Functional Annotation, and Sample Quality Assessment

Eighteen RNA-seq libraries were generated from leaves and roots of *T. coccineum* under control and NaCl treatments (CKL, S100L, S200L, CKR, S100R, and S200R), with three biological replicates per group. After quality filtering, each library yielded more than 38.9 million clean reads and 5.29 Gb of clean sequence data. Q30 values exceeded 98.17% for all libraries, and the mean GC content was 41.19%, indicating high sequencing quality.

**Figure 2:**
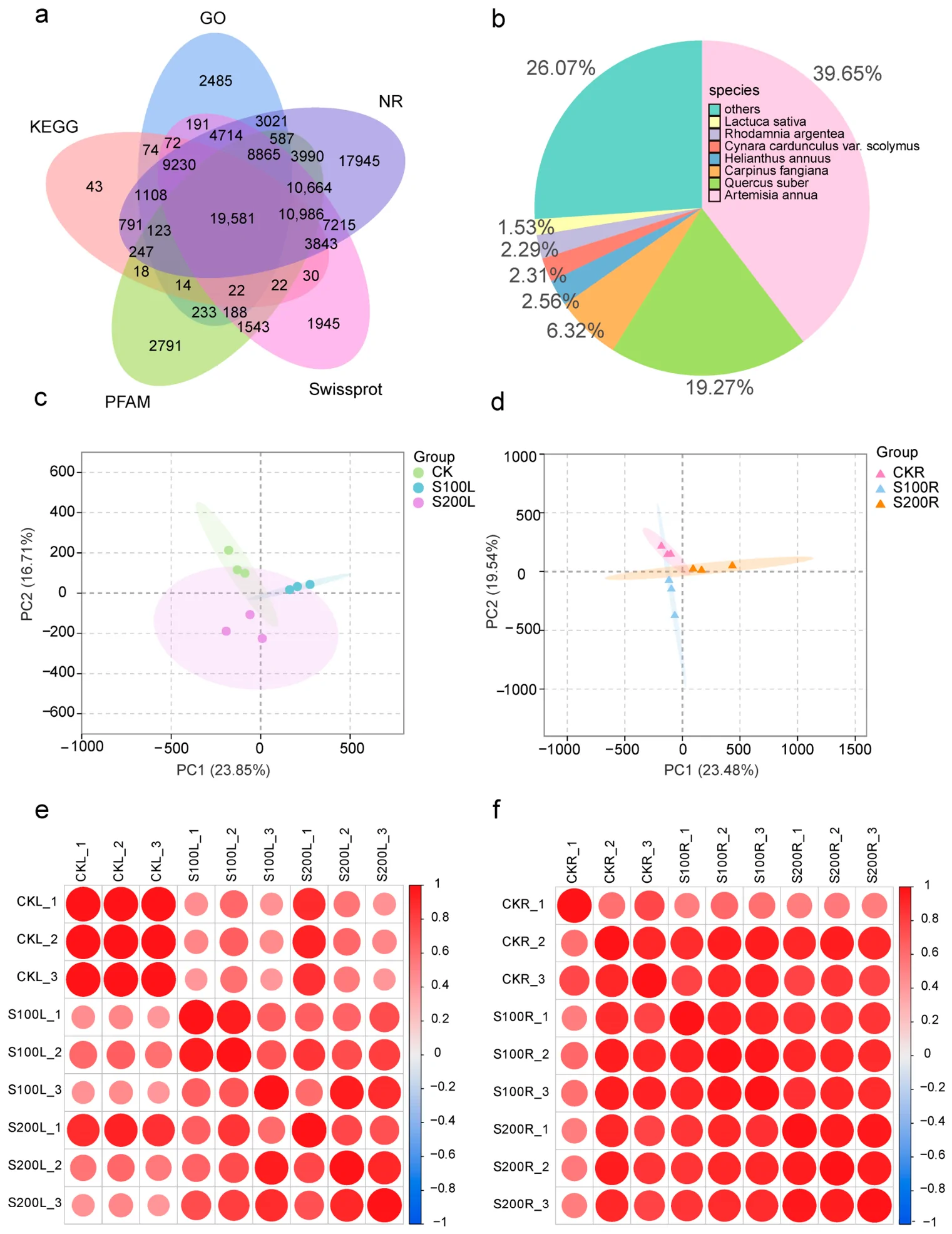
Functional annotation and quality assessment of the *T. coccineum* transcriptome. (**a**) Venn diagram of unigene annotations across five databases; (**b**) species distribution of NR-annotated unigenes; (**c**,**d**) PCA of leaf (**c**) and root (**d**) samples; (**e**,**f**) correlation heatmaps for leaf (**e**) and root (**f**) samples. CKL/R, S100L/R, and S200L/R represent leaves and roots treated with 0, 100, and 200 mM NaCl, respectively.

De novo transcriptome assembly generated 202,517 unigenes, with a mean length of 808.47 bp, an N50 of 1036 bp, and an overall GC content of 41.69%. BUSCO analysis indicated that 80.3% of the expected orthologs were complete. In total, 112,581 (55.59%) unigenes were annotated from at least one of these databases, among which 19,581 (9.67%) unigenes were annotated in all five databases (Figure 2a). The numbers of uniquely annotated unigenes were 17,945 (NR), 2485 (GO), 43 (KEGG), 1945 (Swiss-Prot) and 2791 (Pfam). Among NR-annotated sequences, *Artemisia annua* accounted for the largest proportion of top sequence matches (39.65%), followed by *Quercus suber* (19.27%) and *Carpinus fangiana* (6.32%). Other frequently represented species included *Helianthus annuus* (2.56%), *Cynara cardunculus* var. *scolymus* (2.31%), *Rhodamnia argentea* (2.29%), and *Lactuca sativa* (1.53%), whereas the remaining species were grouped as “others” (26.07%) (Figure 2b).

Subsequent PCA of leaf and root transcriptomes showed that the first two principal components explained 40.56% and 43.02% of the total variance in leaves and roots, respectively (Figure 2c,d). Biological replicates generally clustered within their corresponding treatment groups, whereas the three NaCl treatments were separated in PCA space, indicating treatment-associated differences in transcriptomic profiles. Pearson correlation analysis likewise showed higher correlations among biological replicates within the same treatment than among different treatments (Figure 2e,f). These results collectively indicate good consistency among biological replicates and validated the dataset for subsequent differential expression analyses.

### 2.3. DEG Statistics and Functional Enrichment Under Salt Stress

**Figure 3:**
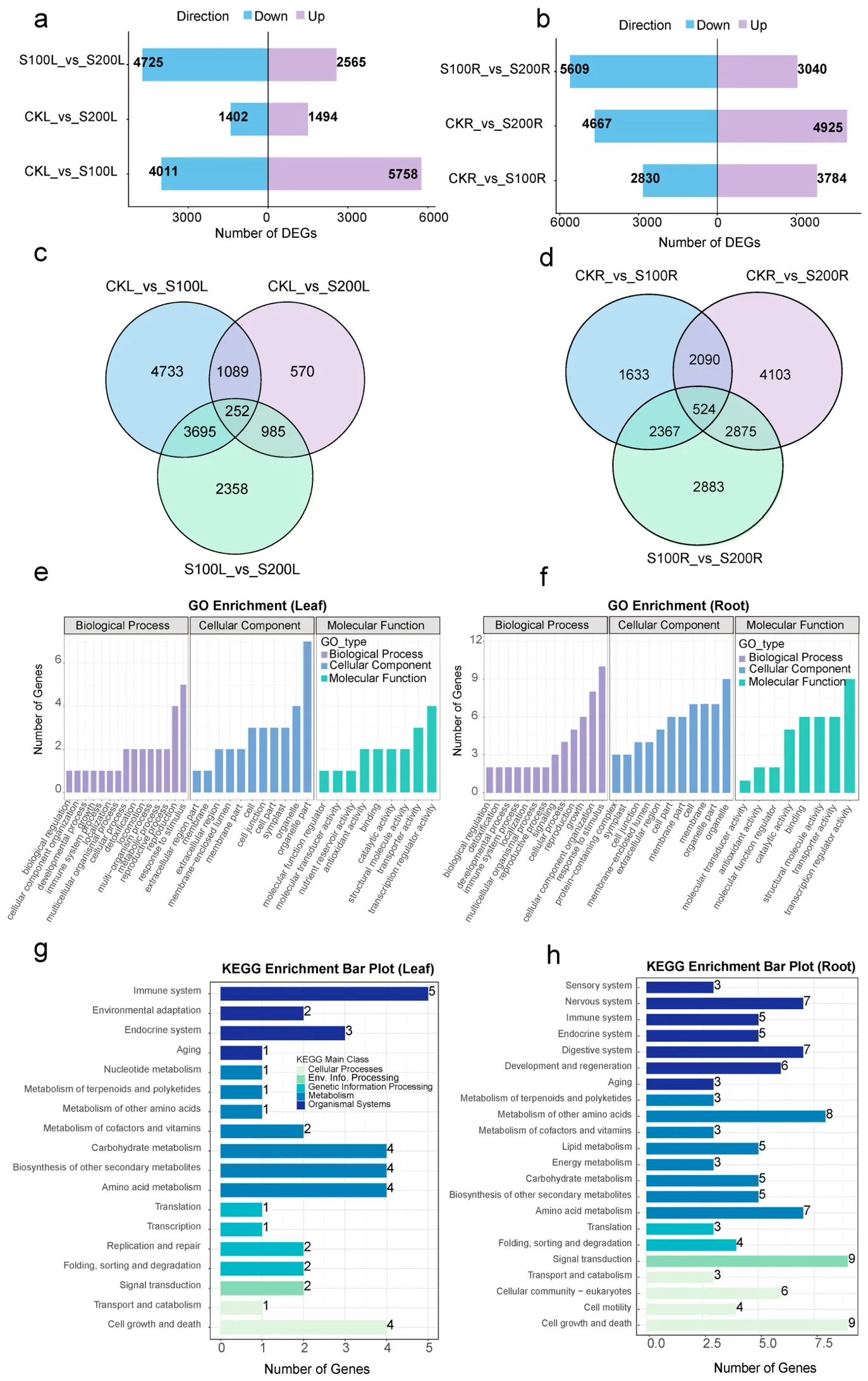
DEGs and functional analysis in leaves and roots of *T. coccineum* under NaCl treatment. (**a**,**b**) Number of upregulated (purple) and downregulated (blue) DEGs in leaves (**a**) and roots (**b**) under different NaCl treatments. CKL/CKR, S100L/S100R, and S200L/S200R represent control, 100 mM NaCl, and 200 mM NaCl treatments in leaves (L) and roots (R), respectively. (**c**,**d**) Venn diagrams showing the overlap of DEGs across treatments in leaves (**c**) and roots (**d**). (**e**,**f**) GO enrichment analysis of DEGs in leaves (**e**) and roots (**f**). (**g**,**h**) KEGG pathway enrichment analysis of DEGs in leaves (**g**) and roots (**h**). The bar length indicates the number of genes enriched in each pathway category.

To characterize transcriptomic changes induced by salt stress, DEGs were identified separately in leaves and roots across three pairwise comparisons among the control, 100 mM NaCl, and 200 mM NaCl treatments (Figure 3a,b). In leaves, 9769 DEGs (5758 upregulated and 4011 downregulated) were detected between S100L and CKL, whereas 2896 DEGs (1494 upregulated and 1402 downregulated) were identified between S200L and CKL. Comparison between S200L and S100L identified 7290 DEGs, including 2565 upregulated and 4725 downregulated genes (Figure 3a). In roots, 6614 DEGs (3784 upregulated and 2830 downregulated) were detected between S100R and CKR, and 9592 DEGs (4925 upregulated and 4667 downregulated) were identified between S200R and CKR. In addition, 8649 DEGs (3040 upregulated and 5609 downregulated) were detected between S200R and S100R (Figure 3b). Notably, the number of DEGs differed markedly between tissues and salt concentrations: leaves had more DEGs than roots at 100 mM NaCl, whereas roots exhibited substantially more DEGs than leaves at 200 mM NaCl.

Venn diagrams were used to examine shared and comparison-specific DEGs across the three pairwise comparisons in leaves and roots (Figure 3c,d). In leaves, 252 DEGs were shared among all three comparisons, whereas substantial numbers of DEGs were specific to individual comparisons (Figure 3c). In roots, 524 DEGs were shared among all three comparisons, together with substantial numbers of DEGs unique to individual pairwise comparisons (Figure 3d).

To explore the potential functions of salt-responsive DEGs, we carried out GO and KEGG enrichment analyses. GO enrichment analysis identified several enriched terms associated with response to stimulus, detoxification, metabolic process, catalytic activity, and transporter activity (Figure 3e,f), suggesting that common transcriptional programs related to stress perception, detoxification, and metabolic adjustment may be activated in both tissues under NaCl treatment. KEGG enrichment analysis further revealed that shared DEGs were assigned to categories including signal transduction, biosynthesis of other secondary metabolites, cell growth and death, amino acid metabolism, and immune-related pathways (Figure 3g,h). Overall, these findings suggest that salt stress elicits both shared and tissue-specific transcriptional responses in *T. coccineum*, primarily involving stress signaling, transport, and primary and secondary metabolism.

### 2.4. WGCNA Module Construction and Functional Annotation

**Figure 4:**
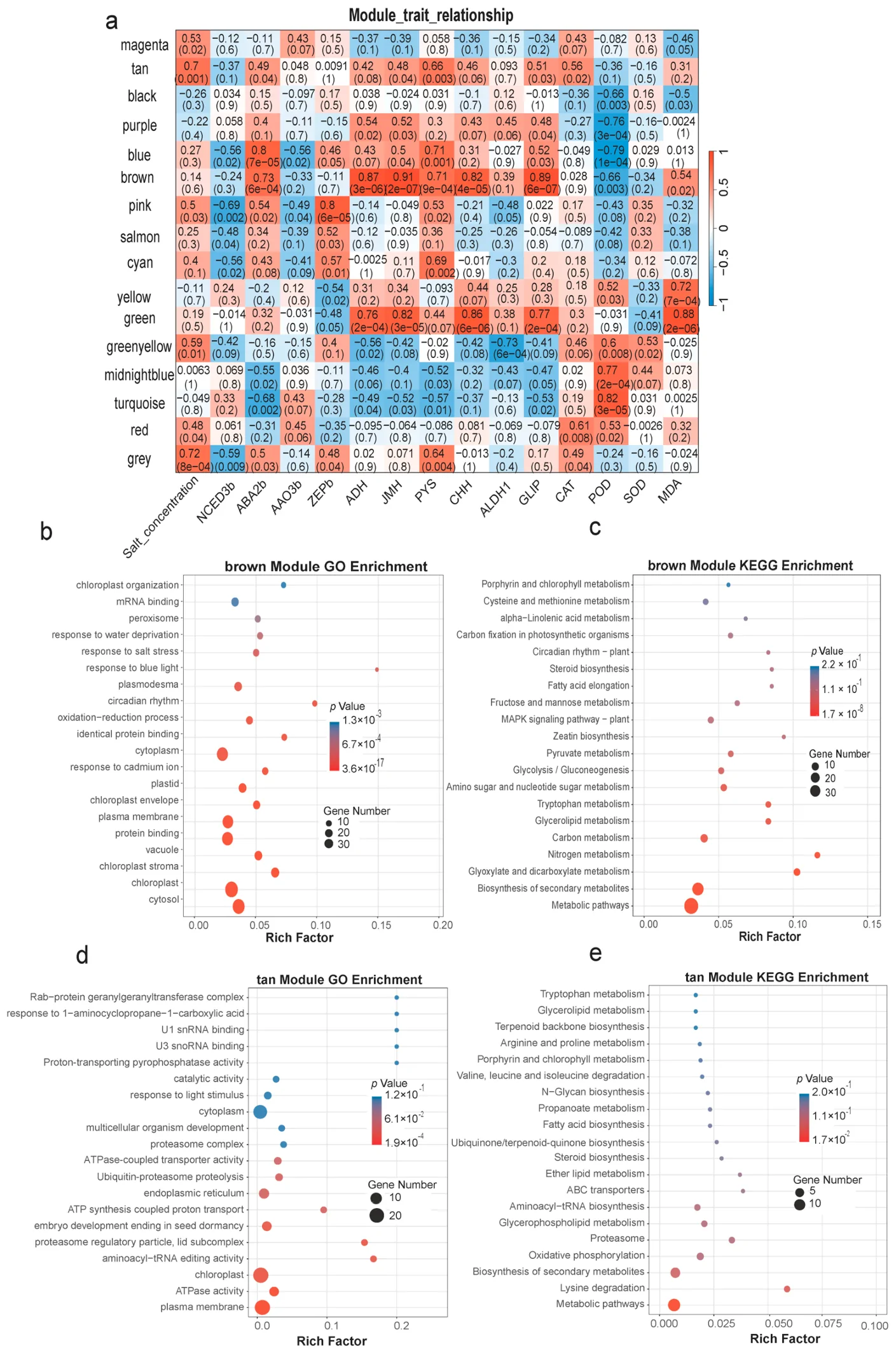
WGCNA module–trait relationships and functional enrichment analyses of the brown and tan modules. (**a**) Correlation heatmap between module eigengenes and salt stress traits. (**b**,**c**) GO and KEGG enrichment analyses of the brown module. (**d**,**e**) GO and KEGG enrichment analyses of the tan module.

To identify co-expression modules associated with salt treatment and salt-responsive physiological, metabolic, and gene expression traits, WGCNA was performed on 15,000 genes retained after expression and variation filtering. A soft-thresholding power of 11 and a merge threshold of 0.3 were applied to construct the network, producing 16 co-expression modules. Two modules, tan (78 genes) and brown (466 genes), displayed distinct correlation patterns with the detected phenotypic and molecular traits and were therefore selected for subsequent functional analysis. The tan module strongly correlated with salt treatment and multiple salt-responsive physiological indicators, suggesting a close association with transcriptional responses to salinity. By contrast, the brown module was positively associated with the transcript abundance of pyrethrin biosynthetic genes, the ABA biosynthesis-related gene *TcABA2b*, and MDA content (Figure 4a). These correlations suggest that the brown module is associated with pyrethrin-related gene expression, *TcABA2b* expression, and lipid peroxidation under salt stress.

We then performed GO and KEGG enrichment analyses to annotate the functional roles of the two modules. In the brown module, enriched GO terms were primarily linked to chloroplast components and stress responses, including salt stress, water deprivation, cadmium ions, and blue light. KEGG analysis further indicated significant enrichment in alpha-linolenic acid metabolism, secondary metabolite biosynthesis, metabolic pathways, and carbon fixation in photosynthetic organisms. In the tan module, enriched GO terms included ATPase activity, ATPase-coupled transmembrane transporter activity, and proteasome-mediated ubiquitin-dependent protein catabolism, while KEGG analysis revealed enrichment in alpha-linolenic acid metabolism, metabolic pathways, arginine and proline metabolism, and secondary metabolite biosynthesis. Taken together, these enrichment patterns suggest that the brown module is primarily linked to stress responses and secondary metabolism, whereas the tan module is associated with transport, protein turnover, and metabolic functions. These functional associations are consistent with distinct components of the salinity response but do not establish direct regulatory roles.

### 2.5. Identification of Candidate Hub Genes and TF-Associated Co-Expression Networks

**Figure 5:**
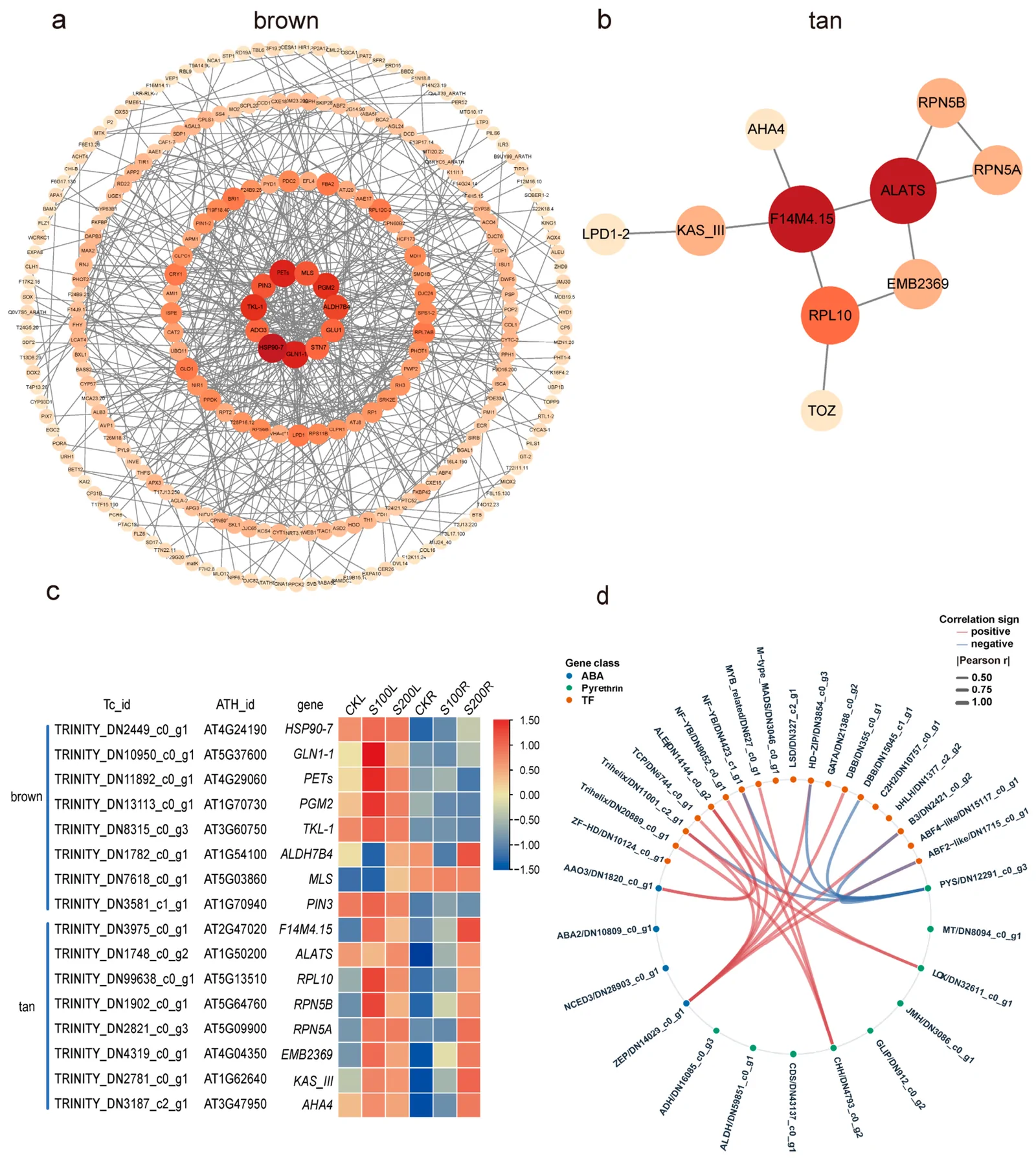
PPI and co-expression networks of candidate genes in the brown and tan modules. (**a**,**b**) PPI networks of candidate hub genes from the brown (**a**) and tan (**b**) modules. For the network nodes, the color depth (from light yellow to dark red) represents the degree of connectivity, with darker red indicating a higher degree. (**c**) Expression heatmap of candidate hub genes. FPKM values were log_2_-transformed and row-normalized. (**d**) Tissue-adjusted TF-centered co-expression network. Orange, blue, and green nodes represent TFs, ABA-related genes, and pyrethrin-related genes, respectively. Red and blue edges indicate positive and negative correlations, respectively, with thickness representing the absolute partial correlation coefficient. Only significant associations are shown (BH-adjusted *p* ≤ 0.05).

PPI networks were constructed for genes in the brown and tan modules based on their *A. thaliana* homologs. Genes were ranked according to degree centrality, and the eight highest-ranking nodes in each network were identified as candidate hub genes for subsequent expression profiling (Figure 5a–c).

Several candidate hub genes in the brown module were annotated as being related to stress response and metabolic adjustment. The highly connected candidate hub *ALDH7B4* (aldehyde dehydrogenase 7B4) encodes an aldehyde dehydrogenase that has been associated with aldehyde detoxification and oxidative stress responses in plants [21], suggesting a possible role in mitigating salt-induced oxidative damage (Figure 5a). *TKL-1* (transketolase-1) and *PGM2* (phosphoglucomutase 2) are involved in primary carbon metabolism and may contribute to the supply or redistribution of metabolic intermediates under salt stress. *GLN1-1* (glutamine synthetase 1;1) is associated with nitrogen assimilation, whereas *HSP90-7* (heat shock protein 90-7) encodes a molecular chaperone that may participate in protein stabilization under stress conditions. Other hub genes, including *PETs* (plastid-specific ribosomal protein 7), *MLS* (malate synthase) and *PIN3* (auxin efflux carrier component 3), were annotated as being related to photosynthetic processes, organic acid metabolism, or hormone transport. Taken together, these annotations suggest that the brown module may contribute to oxidative stress response, primary metabolic adjustment, and stress-related metabolic regulation under salinity, but their direct roles in ABA metabolism or pyrethrin biosynthesis require further validation.

In the tan module, *AHA4* (plasma membrane H^+^-ATPase 4), *RPN5A* (26S proteasome regulatory subunit RPN5a) and *RPN5B* (26S proteasome regulatory subunit RPN5b) were among the highly connected candidate hub genes. *AHA4* encodes a plasma membrane H^+^-ATPase and may be associated with ion transport and cellular ion homeostasis under salt stress. *RPN5A* and *RPN5B* encode regulatory subunits of the 26S proteasome, suggesting that protein turnover is potentially associated with the salt-stress response of *T. coccineum*. Other candidate hub genes, such as *KAS_III* (3-ketoacyl-acyl carrier protein synthase III), *F14M4.15* (peptide chain release factor 1), *ALATS* (alanine-tRNA ligase), *RPL10* (50S ribosomal protein L10) and *EMB2369* (leucine-tRNA ligase), were mainly annotated as being related to lipid metabolism, translation, or plant development. These results suggest that the tan module may be linked to ion homeostasis, protein metabolism, and general cellular adjustment under salt stress.

Expression heatmap analysis showed that, in the brown module, *ALDH7B4* and *MLS* were detected in both roots and leaves; in leaves, their expression was detected only under the 200 mM NaCl treatment. The other candidate hub genes were expressed predominantly in leaves and generally showed their highest relative expression at 100 mM NaCl. In the tan module, all candidate hub genes were detected in both roots and leaves. Most showed relatively high expression in leaves under 100 or 200 mM NaCl, whereas their relative expression in roots was generally highest at 200 mM NaCl (Figure 5c).

To further explore the potential associations between the selected modules and ABA- and pyrethrin-related pathways, 18 putative TFs belonging to 14 families were identified in the brown module, whereas only one bHLH TF was identified in the tan module. Among the brown-module TFs, two bZIP genes were annotated as ABF2-like and ABF4-like. Tissue-adjusted Pearson partial correlation analysis identified 20 significant associations among 247 possible pairs between the 19 TFs and 13 ABA- or pyrethrin-related genes (BH-adjusted *p* ≤ 0.05; Figure 5d). The network included both positive and negative correlations. Notably, ABF2-like was positively correlated with *ZEP* (*r* = 0.796), whereas a B3-family TF showed a positive correlation with *ZEP* (*r* = 0.796) but a negative correlation with *PYS* (*r* = −0.761). A Trihelix TF was also positively correlated with *CHH* (*r* = 0.809). These findings reveal distinct co-expression patterns between candidate TFs and ABA- or pyrethrin-related genes, suggesting their potential involvement in transcriptional responses to salt stress.

Together, the PPI and TF-centered co-expression analyses identified candidate hub genes and potential transcriptional associations related to salt-stress responses and ABA- and pyrethrin-related metabolism. However, these network-based associations do not establish direct regulatory interactions and require further experimental validation.

### 2.6. Salt Stress Alters the Expression of ABA Biosynthesis-Related Genes

**Figure 6:**
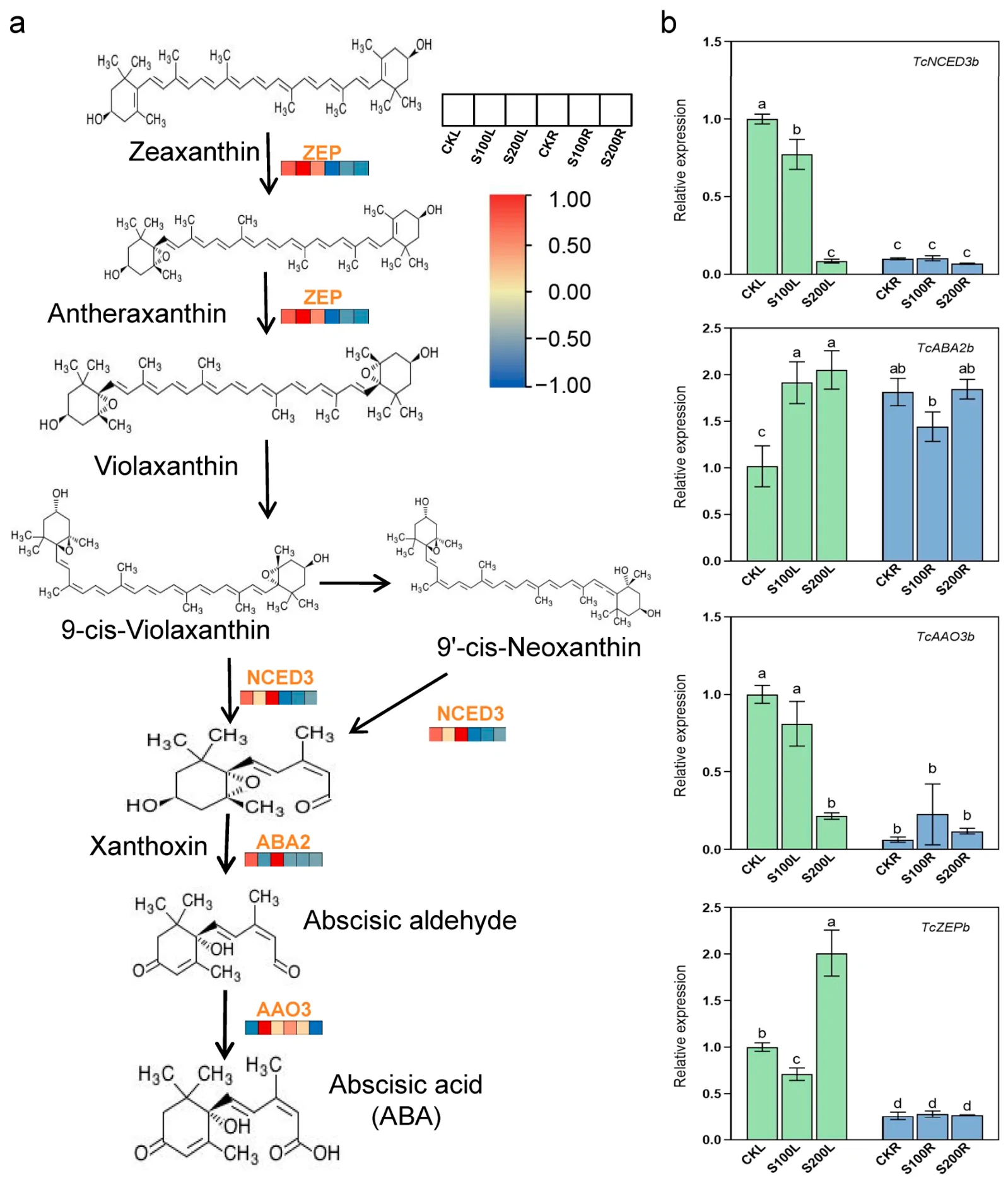
ABA biosynthetic pathway and expression of ABA biosynthesis-related genes. (**a**) Schematic diagram of the abscisic acid (ABA) biosynthetic pathway. Gene expression data were log_2_ (+1) transformed and row-normalized. The color bar represents normalized values ranging from −1 to 1; red and blue indicate relatively high and low expression, respectively. (**b**) Relative expression of ABA biosynthetic genes (*TcABA2b*, *TcAAO3b*, *TcZEPb*, *TcNCED3b*) measured by RT-qPCR. Green and blue bars correspond to leaf and root samples, respectively. Expression levels were normalized to the leaf control (CKL), and values are presented as mean ± SD (*n* = 3). CKL/R, control (0 mM NaCl); S100L/R, 100 mM NaCl treatment; S200L/R, 200 mM NaCl treatment. L, leaf; R, root. Different lowercase letters denote significant differences among treatments based on one-way ANOVA followed by Tukey’s HSD test (*p* < 0.05).

To examine the transcriptional response of the ABA biosynthetic pathway to salinity, four genes encoding key enzymes in ABA biosynthesis were selected for further analysis: *TcZEPb* (*TRINITY_DN14029_c0_g1*), *TcNCED3b* (*TRINITY_DN28903_c0_g1*), *TcABA2b* (*TRINITY_DN10809_c0_g1*), and *TcAAO3b* (*TRINITY_DN1820_c0_g1*) (Figure 6a). Their expression patterns were further examined by RT-qPCR (Figure 6b). The four genes exhibited distinct responses to NaCl treatment in leaves. *TcNCED3b* (9-cis-epoxycarotenoid dioxygenase 3) and *TcAAO3b* (abscisic aldehyde oxidase 3) transcript levels decreased progressively with increasing NaCl concentration, whereas *TcABA2b* (abscisic acid deficient 2) expression increased with increasing salinity. *TcZEPb* (zeaxanthin epoxidase) expression decreased at 100 mM NaCl and subsequently increased at 200 mM NaCl. In roots, the overall transcriptional responses of these genes were less pronounced than those observed in leaves. *TcABA2b* showed a significant response to NaCl treatment, whereas *TcZEPb*, *TcNCED3b*, and *TcAAO3b* remained at relatively low expression levels and showed comparatively limited responses to NaCl treatment.

Overall, the four ABA biosynthesis-related genes displayed distinct, tissue-dependent transcriptional responses to salinity, with more pronounced changes observed in leaves than in roots. Because endogenous ABA concentrations were not measured, these transcriptional changes should not be taken as direct evidence of altered ABA accumulation.

### 2.7. Pyrethrin Biosynthesis-Related Gene Expression and Accumulation Under Salt Stress

**Figure 7:**
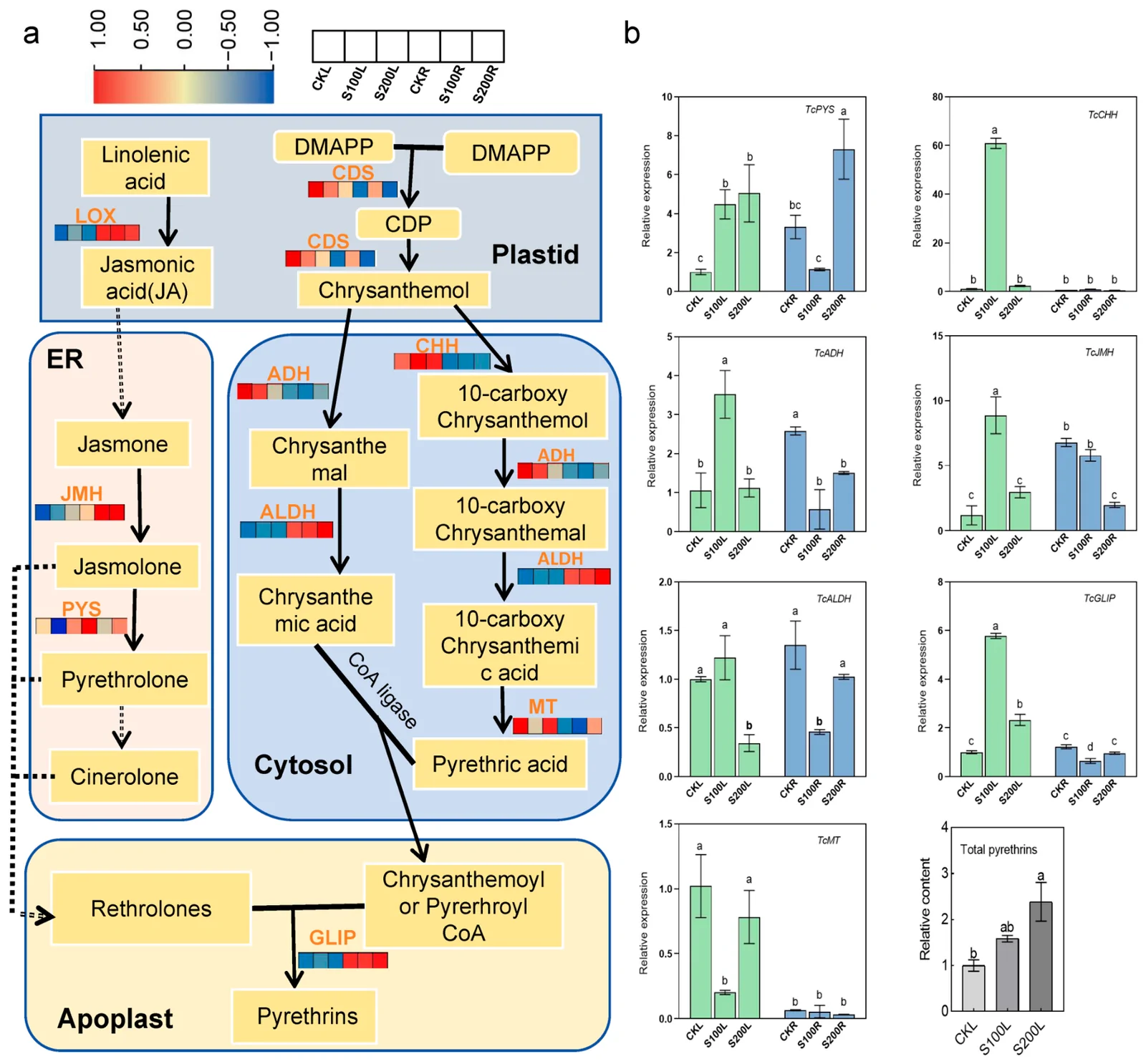
Pyrethrin biosynthetic pathway, gene expression and leaf pyrethrin content under salt stress. (**a**) Schematic of the pyrethrin biosynthetic pathway. Gene expression data were log_2_ (+1) transformed and row-normalized. The solid arrows indicate the experimentally confirmed enzymatic steps where genes/enzymes have been identified, while the dashed arrows represent putative or uncharacterized steps requiring further experimental validation. The color bar represents normalized values ranging from −1 to 1; red and blue indicate relatively high and low expression, respectively. (**b**) RT-qPCR results of pyrethrin biosynthetic genes and leaf pyrethrin content. Data are presented as means ± SD of three biological replicates. CKL/R: 0 mM NaCl; S100L/R: 100 mM NaCl; S200L/R: 200 mM NaCl; L = leaf, R = root. All leaf and root samples were included in a single one-way ANOVA model. Significant differences were evaluated by one-way ANOVA followed by Tukey’s HSD test (*p* < 0.05), marked with different lowercase letters.

The known pyrethrin biosynthetic pathway in *Tanacetum* species consists of two major branches: the acid-moiety branch, which provides chrysanthemic or pyrethric acid derivatives, and the alcohol-moiety branch, which provides rethrolone compounds (Figure 7a). To examine the transcriptional responses of this pathway to salinity, the expression patterns of genes annotated as being associated with pyrethrin biosynthesis were analyzed. RNA-seq expression profiles showed marked differences between leaf and root tissues. Multiple genes related to the acid-moiety branch, including *TcCDS* (chrysanthemyl diphosphate synthase), *TcADH* (alcohol dehydrogenase), *TcMT* (10-carboxychrysanthemic acid 10-methyltransferase), and *TcCHH* (chrysanthemol 10-hydroxylase), showed relatively higher transcript abundance in leaves, whereas *TcALDH* (aldehyde dehydrogenase) and *TcGLIP* (GDSL lipase-like protein) exhibited relatively higher expression in roots. Several genes associated with the alcohol-moiety branch also showed higher relative transcript abundance in roots.

RT-qPCR was subsequently used to examine the expression patterns of seven selected pyrethrin-related genes, including *TcPYS* (pyrethrolone synthase), *TcADH*, *TcALDH*, *TcCHH*, *TcMT*, *TcJMH* (jasmone hydroxylase), and *TcGLIP* (Figure 7b). In leaves, *TcPYS* expression increased with increasing NaCl concentration, whereas *TcMT* expression decreased at 100 mM NaCl and increased again at 200 mM NaCl. The remaining tested genes generally showed higher transcript levels at 100 mM NaCl than at 200 mM NaCl, indicating heterogeneous and non-linear transcriptional responses of pyrethrin biosynthesis-related genes to increasing salinity. In roots, most of the tested genes showed relatively low transcript abundance at 100 mM NaCl, with varying expression responses at 200 mM NaCl. Pyrethrins were not detected in root extracts under the present assay conditions; therefore, pyrethrin content was evaluated only in leaves. Leaf pyrethrin content increased with NaCl concentration (Figure 7b). Thus, although individual pyrethrin biosynthesis-related genes exhibited distinct transcriptional responses to salinity, increased NaCl concentration was associated with higher estimated pyrethrin content in leaves.

The expression trends of these genes detected by RT-qPCR were generally consistent with transcriptomic data, supporting the reliability of our RNA-seq results.

## 3. Discussion

### 3.1. Effects of Salt Stress on Physiological and Biochemical Characteristics of T. coccineum

Salt stress can cause oxidative damage and affect plant growth [22]. In this study, *T. coccineum* showed increasing leaf injury with NaCl concentration, with chlorosis observed at 100 mM and necrosis at 200 mM. Similar responses have been reported in other salt-sensitive plants including *Juglans microcarpa* and *Nicotiana tabacum* [23,24]. The increase in CAT activity in both leaves and roots suggests an important role for CAT-mediated H_2_O_2_ scavenging during salt stress, consistent with previous findings in *Coreopsis tinctoria* and *Brassica napus* under salt stress [25,26]. In contrast, SOD and POD activities exhibited divergent patterns between leaf and root tissues. Roots are directly exposed to salinity, and the changes in SOD and POD activities may reflect adjustments of ROS-scavenging capacity upon salt exposure. Similar tissue-dependent antioxidant responses have been reported in *Taxodium mucronatum* [27]. These findings indicate enzyme- and organ-specific changes in antioxidant activity under saline conditions.

MDA content increased at 100 mM NaCl but decreased at 200 mM in both tissues. Similar non-linear changes have been reported in *Malus* species [28]. Although MDA is commonly used as an indicator of membrane lipid peroxidation [29,30], its decrease at 200 mM did not correspond to reduced visible injury. This suggests that MDA content alone may not fully reflect the severity of salt-induced damage. Overall, these findings indicate that antioxidant responses in *T. coccineum* depend on both tissue type and salt concentration.

### 3.2. Functional Roles of Candidate Hub Genes in the Tan and Brown Modules

WGCNA identified the tan and brown modules as being associated with different aspects of salt-stress responses in *T. coccineum*. The tan module was mainly associated with ion transport, cellular organization, membrane-related functions, and protein metabolism, whereas the brown module was linked to stress responses and secondary metabolism. PPI analysis further identified several candidate hub genes with potential roles in these processes.

In the tan module, *AHA4* belongs to the P-type plasma membrane H^+^-ATPase family, whose members establish transmembrane proton gradients that support ion homeostasis and metabolite transport during salt stress [31]. In *Arabidopsis*, *AHA4* is localized in root endodermal cells, where it contributes to salt-stress response by maintaining ion balance. Disruption of *AHA4* leads to a 4–5-fold rise in the leaf Na^+^/K^+^ ratio under salt stress [32], and the proton gradient established by *AHA4* drives the Na^+^/H^+^ antiporter *SOS1* to remove Na^+^ from the cytosol [33]. The activity of *AHA4* is negatively regulated by the calcium sensor *CBL10* under alkaline stress [34], and a study in *Salix linearistipularis* reported that *AHA4* overexpression enhanced root H^+^ efflux and reduced Na^+^ accumulation, thereby improving salt-stress response [35]. Similarly, the Arabidopsis *AHA2* is also activated under salt stress, and the resulting proton motive force facilitates Na^+^ efflux to enhance salt resistance [36]. These observations support the possibility that the AHA4-like candidate identified here may be associated with ion homeostasis under salt stress, although its specific function in *T. coccineum* remains to be experimentally verified. *RPN5A* and *RPN5B* encode paralogous non-ATPase subunits of the 19S regulatory particle of the 26S proteasome, and *RPN5A* is the primary contributor to proteasome assembly and stability, whereas *RPN5B* is partially redundant [37]. As hub genes identified in the tan module, their upregulation under salt stress may reflect enhanced proteasome-mediated protein turnover required for stress adaptation. Other candidate hub genes were annotated with functions related to lipid metabolism, translation, and plant development, suggesting additional components of cellular adjustment under salt stress.

In the brown module, *ALDH7B4* has been implicated in ABA-related and oxidative-stress responses in several plant species. Evidence from multiple taxa supports a conserved stress-related role. In the halophyte *Eutrema salsugineum*, *EsALDH7B4* is strongly activated by salt and ABA in a promoter-dependent manner [38]. *AtALDH7B4* in *A. thaliana* contributes to the detoxification of lipid peroxidation products and to reduced MDA and ROS accumulation, thereby alleviating oxidative damage [21,39]. *ALDH7B4* has also been associated with secondary metabolic regulation in *Gossypium hirsutum,* where *GhALDH7B4* promotes lignin and polysaccharide biosynthesis [40]. Related stress-responsive expression has also been reported in woody and horticultural species: *PaALDH17* in *Prunus avium* is the most strongly induced ALDH member under saline-alkali stress and enhances salinity tolerance via antioxidant activation [41], and salt-inducible *ALDH7B4* expression has also been observed in *Fragaria × ananassa* [42]. Collectively, these findings support a conserved stress-related role for *ALDH7B4* in detoxification, whereas its contribution to secondary metabolism may be context dependent. The ALDH superfamily also shows substantial functional diversification. In pyrethrum, *TcALDH1* catalyzes a committed step in pyrethrin biosynthesis, oxidizing *trans*-chrysanthemal to *trans*-chrysanthemic acid [43], yet its promoter similarly harbors ABA-responsive elements and is induced by MeJA and ABA [44]. These observations suggest that stress-responsive ALDH genes and pyrethrin biosynthesis-related ALDH genes may both be influenced by hormone-responsive regulatory pathways. In addition, *TKL-1* and *PGM2* may link primary carbon metabolism with secondary metabolite biosynthesis. *TKL-1*, a pentose phosphate pathway enzyme, generates E4P (erythrose-4-phosphate) for the shikimate pathway, supplying carbon skeletons for phenylpropanoid-derived metabolites [45]. A recent study in *Petunia* further demonstrates that transketolase provides the primary *E4P* source for anthocyanin biosynthesis, underscoring its general importance in channeling carbon skeletons into diverse secondary metabolic pathways [46]. Similarly, *PGM2* can influence carbon flux toward UDP-glucose pools, which provide glycosyl donors for secondary metabolite glycosylation [47]. Their presence among highly connected genes in the brown module suggests that carbon and precursor metabolism may be associated with the salt-responsive network.

### 3.3. Potential Transcriptional Associations Between ABA and Pyrethrin-Related Metabolism Under Salt Stress

ABA biosynthesis-related genes exhibited distinct transcriptional responses to salt stress in *T. coccineum*. In leaves, *TcABA2b* expression increased with NaCl concentration, whereas *TcNCED3b* and *TcAAO3b* decreased, and *TcZEPb* showed a non-linear response. These changes were generally less pronounced in roots, indicating tissue-dependent differences in the transcriptional responses of individual ABA biosynthetic steps. Several pyrethrin biosynthesis-related genes also showed increased expression under moderate salinity (S100L), although their responses were less uniform at 200 mM NaCl. Meanwhile, spectrophotometrically estimated leaf pyrethrin content increased with NaCl concentration. In *T. cinerariifolium*, pyrethrins accumulate predominantly in flower heads, with substantially lower levels in leaves, and the pathways supplying their precursors exhibit tissue-dependent expression [48]. The changes observed in *T. coccineum* leaves therefore suggest a potential response of pyrethrin-related metabolism to salinity. The incomplete correspondence between transcript abundance and estimated metabolite content may reflect differences between transcriptional responses and cumulative metabolite accumulation, which depends on biosynthesis, turnover, and transport. Similar differences between biosynthetic gene expression and pyrethrin accumulation have been reported during flower development in *T. coccineum* [4], and *T. cinerariifolium* [49]. However, the single sampling time used in the present study does not allow these temporal relationships to be resolved.

A GLIP-like transcript also exhibited relatively high expression in roots. Biochemical characterization of *TcGLIP* in *T. cinerariifolium* demonstrated both acyltransferase and esterase activities, with the former predominating under the tested conditions [50]. Subsequent structural analyses suggested that specific amino acid residues and substrate-binding properties contribute to the transferase activity of GDSL lipases [51]. Nevertheless, pyrethrin biosynthesis requires the formation of monoterpenoid acid and oxylipin-derived alcohol precursors through multiple enzymatic reactions before their final esterification [52]. These processes involve distinct subcellular compartments and exhibit pronounced spatial organization in floral tissues [53]. Consequently, elevated GLIP-like transcript abundance in roots does not necessarily indicate substantial pyrethrin biosynthesis, because precursor availability, upstream enzymatic activities, and the catalytic properties of the encoded protein may constrain the pathway.

Further analysis identified 19 candidate TFs in the brown and tan modules. A tissue-adjusted co-expression network revealed significant associations between these TFs and ABA- or pyrethrin-related genes. The presence of both positive and negative correlations suggests that these candidate TFs may participate in the regulation of different biosynthetic steps rather than uniformly activating the two pathways. In *T. cinerariifolium*, the ABA- and MeJA-responsive transcription factor *TcbZIP60* has been shown to directly activate pyrethrin biosynthesis-related genes [16], supporting the potential involvement of hormone-responsive TFs in pyrethrin metabolism. Moreover, ABA-dependent transcriptional regulation can be modulated by protein phosphorylation, as demonstrated by the phosphorylation of *AabZIP1* by *AaAPK1* during artemisinin biosynthesis in *Artemisia annua* [54]. These findings suggest that the candidate TFs identified here may contribute to salt-responsive metabolic regulation, although their regulatory activities and direct target genes remain to be experimentally established.

Nevertheless, the regulation of specialized metabolite accumulation under abiotic stress is considerably more complex than the transcriptional activation of individual biosynthetic genes, particularly for compounds synthesized through multiple metabolic pathways. Although drought and salt stress commonly trigger ABA-related responses, their effects on specialized metabolism may differ depending on stress intensity, duration, and plant developmental status. In *A. annua*, the ABA-responsive *AabZIP1* directly activates the JA-signaling regulator *AaMYC2*, which subsequently promotes the expression of downstream artemisinin biosynthesis genes, demonstrating an indirect regulatory connection between ABA and specialized metabolism. The same study also highlighted that short-term water deprivation or ABA treatment can promote artemisinin biosynthesis, whereas prolonged drought reduces plant biomass and artemisinin production [55]. Similar complexity has been observed in pyrethrum. Dryland conditions have been shown to reduce pyrethrin yield by affecting flower, achene, and glandular trichome development [56]. In contrast, exogenous ABA increased pyrethrin II accumulation in *T. cinerariifolium* seedlings, while PEG and NaCl treatments also promoted the accumulation of this compound [57]. However, reduced pyrethrin accumulation has been reported under PEG-induced drought stress despite ABA-related transcriptional responses [20]. These contrasting observations indicate that ABA-related stress responses do not necessarily result in consistent changes in pyrethrin accumulation. The relationship between salt-induced ABA responses and pyrethrin metabolism in *T. coccineum* may therefore involve multiple interacting regulatory and metabolic processes rather than a single ABA-dependent transcriptional mechanism.

## 4. Materials and Methods

### 4.1. Plant Cultivation and Treatment

*T. coccineum* seeds obtained from our lab were used for all experiments. Seeds exhibiting wrinkling, mechanical injury, or other visible defects were excluded prior to sowing. The remaining viable seeds were sown in seedling trays filled with a 2:1 (*v*/*v*) mixture of Pindstrup peat (0–6 mm particle size, pH 5.5–6.0, EC 1.0–1.5 mS cm^−1^) and vermiculite. Plants were cultivated at 25 °C under a 14 h light/10 h dark photoperiod with 70% relative humidity (RH). At the 3–4 true-leaf stage, seedlings of uniform size were individually transplanted into plastic pots (12 cm diameter). After a 7-day acclimatization period, salt treatments were initiated.

To determine suitable NaCl concentrations for the main experiment, a preliminary trial was conducted using 0, 100, 150, 200, and 250 mM NaCl under identical cultivation conditions described above. Each treatment comprised three biological replicates, with one independently grown seedling serving as one replicate. Following 7 days of salt treatment, plant injury was evaluated visually based on leaf chlorosis, wilting and necrosis. Seedlings exposed to 250 mM NaCl exhibited severe injury and mortality within 7 days, precluding sampling and subsequent multi-index analysis. The 150 mM treatment produced intermediate phenotypic responses between 100 mM and 200 mM NaCl and did not provide a clearly distinguishable stress level. Therefore, 0, 100, and 200 mM NaCl were selected for the main experiment to represent different levels of salt stress.

In the main experiment, seedlings were treated with 0, 100, or 200 mM NaCl; control plants received deionized water, whereas the two salt-treatment groups received NaCl solutions prepared with deionized water. Each treatment comprised three biological replicates, with one independently grown plant representing one biological replicate. Each pot received 200 mL of the corresponding solution every 2 days, with the same irrigation volume and frequency across treatments. After 7 days of treatment, whole-plant phenotypes were photographed, and leaves and roots were harvested separately. The collected samples were immediately frozen in liquid nitrogen and stored at −80 °C until physiological and transcriptomic analyses.

### 4.2. Assays of Antioxidant Enzyme Activities, MDA, and Pyrethrin Content

Fresh leaves and roots were analyzed separately for SOD, POD, and CAT activities and malondialdehyde (MDA) content using commercial assay kits (Beijing Solarbio Science & Technology Co., Ltd., Beijing, China; Cat. Nos. BC0170, BC0090, BC0200, and BC0020, respectively), following the manufacturer’s protocol. SOD activity was determined based on the xanthine/xanthine oxidase (XOD)-NBT system (560 nm); POD activity was measured by monitoring the H_2_O_2_-mediated oxidation of specific substrates (470 nm); CAT activity was assayed via the decrease in H_2_O_2_ absorbance (240 nm); and MDA content was quantified using the thiobarbituric acid (TBA) method, with absorbance measured at both 532 and 600 nm to correct for background turbidity. Enzyme activities were expressed as U g^−1^ fresh weight (FW), and MDA content as nmol g^−1^ FW. Approximately 0.10 g of fresh tissue was used for each assay, with three independent biological replicates for each treatment and tissue. Leaf pyrethrin content was estimated using the rapid spectrophotometric method previously described [58]. Leaf tissues were initially dried at 50 °C to constant weight and then ground into a fine powder. A 0.10 g portion of the powder was transferred to a centrifuge tube and extracted with 5 mL of n-hexane. The extraction was performed by sequential vortex-mixing (30 s), sonication (40 kHz, 10 min), and re-vortexing (30 s), after which the mixture was centrifuged at 1300× *g* for 6 min at 25 °C. An aliquot of the supernatant (0.5 mL) was diluted with 9.5 mL of anhydrous ethanol, and the absorbance of the resulting solution was recorded at 225 nm using a FlexA-200 microplate reader (Hangzhou Allsheng Instruments Co., Ltd., Hangzhou, Zhejiang, China). This wavelength corresponds to the characteristic UV absorption maximum of natural pyrethrins reported in previous studies [59,60]. Because this spectrophotometric method does not chromatographically resolve individual pyrethrin esters, the resulting values were interpreted as estimates of total pyrethrin content and expressed on a dry-weight basis. Three independent biological replicates were analyzed for each treatment, with two analytical measurements per biological replicate. The mean value of the two measurements for each plant was used for subsequent data analysis.

### 4.3. RNA Extraction and Sequencing

Total RNA was extracted from leaves and roots of *T*. *coccineum* seedlings using TRIzol reagent (Invitrogen, Waltham, MA, USA, Cat. No. 15596026) following the manufacturer’s instructions. High-quality RNA samples were sent to a commercial sequencing provider (Personalbio Biotechnology, Shanghai, China) for library construction and paired-end (150 bp) sequencing on a DNBSEQ-T7 platform (BGI, Shenzhen, Guangdong, China). Raw reads were filtered by fastp v0.22.0 [61]. The obtained clean reads were assembled de novo into unigenes using Trinity v2.6.6 [62], which served as the reference transcriptome for downstream analyses. BUSCO v5.8.3 was used to evaluate the assembly completeness [63]. Clean reads from each library were mapped to the assembled reference transcriptome using Bowtie2 v2.3.4 [64], and transcript abundance was quantified using RSEM v1.3.1 [65]. Pearson correlation and principal component analysis (PCA) were completed on the LC-BIO cloud platform to assess consistency among biological replicates.

DESeq2 v1.20 was used to identify DEGs with thresholds of |log_2_FoldChange| > 1 and adjusted *p*-value < 0.05 [66]. All unigenes were annotated against NR, Swiss-Prot, Pfam, KEGG, and GO databases, followed by GO and KEGG enrichment analyses of DEGs using KOBAS v3.0 [67].

### 4.4. WGCNA and PPI Analyses

Genes with an average FPKM > 1 and a coefficient of variation (CV) > 0.3 were included in WGCNA. A minimum gene number of 50 was set for module construction. Correlations between module eigengenes and experimental traits were examined to detect module–trait associations, and modules strongly associated with salt-responsive traits were retained for further analysis. GO and KEGG enrichment analyses of genes in selected modules were performed using the procedures described above.

PPI networks for genes in salt stress-associated modules were constructed using the STRING database (http://string-db.org/) and visualized using Cytoscape v3.10.4. Genes with high network connectivity were prioritized as candidate hub genes. Transcription factors (TFs) were identified using PlantTFDB 4.0 (https://planttfdb.gao-lab.org/). Based on functional annotations, four ABA biosynthesis-related genes and eight pyrethrin biosynthesis-related genes were selected. Tissue-adjusted Pearson partial correlations were calculated using log_2_(FPKM + 1)-transformed expression data from 18 RNA-seq samples, with tissue effects removed by linear regression. *p*-values were adjusted using the Benjamini–Hochberg method. Significant correlations (adjusted *p* < 0.05) were used to construct a co-expression network.

### 4.5. Validation by RT-qPCR

To validate the RNA-seq data, eleven genes were chosen for RT-qPCR analysis, including four associated with ABA biosynthesis and seven involved in pyrethrin biosynthesis. Total RNA was extracted from the same plant samples used for transcriptome sequencing, and cDNA was synthesized with the All-in-One First-Strand Synthesis MasterMix kit (Yugong Biotechnology, Wuxi, China). RT-qPCR was carried out on an FQD-96A system (Bioer Technology, Hangzhou, China) with Taq SYBR^®^ Green qPCR Premix (Universal) (Yugong Biotechnology, Wuxi, China). Each 20 µL reaction contained 1 µL of cDNA template, 1 µL of each primer, 10 µL of SYBR Green Premix, and 7 µL of nuclease-free water, with *TcGAPDH* as the reference gene [4]. Gene-specific primers were designed using TBtools-II v2.454, with sequences listed in Table S1. The amplification conditions were as follows: 95 °C for 30 s, followed by 40 cycles at 95 °C for 10 s and 60 °C for 10 s. To ensure amplicon specificity and rule out primer-dimers, a melting curve was included. Relative expression was quantified using the 2^−ΔΔCt^ method [68], with three biological and two technical replicates.

### 4.6. Statistical Analysis

Statistical analyses were conducted with R v4.2.1. Group comparisons were performed by one-way ANOVA, with Tukey’s HSD test for multiple comparisons. Statistical significance was defined as *p* < 0.05.

## 5. Conclusions

This study integrated physiological measurements, transcriptome profiling, and network analysis to investigate tissue-specific responses to salt stress and changes in pyrethrin-related metabolism in *T. coccineum*. Increasing NaCl concentrations intensified leaf injury and induced distinct antioxidant responses in leaves and roots. Pyrethrin biosynthesis-related genes exhibited variable transcriptional responses to salinity, whereas spectrophotometrically estimated leaf pyrethrin content increased with NaCl concentration. ABA biosynthesis-related genes also showed tissue-dependent transcriptional responses. WGCNA identified the tan and brown modules, which were associated with salt-responsive processes and secondary metabolism, respectively. Candidate hub genes, including *AHA4* and *ALDH7B4*, were identified, while subsequent co-expression analysis revealed potential associations between candidate TFs and ABA- or pyrethrin-related genes. Collectively, these findings provide insights into tissue-specific salt-stress responses and the transcriptional complexity of pyrethrin-related metabolism in *T. coccineum*, providing a basis for further functional studies.

## Data Availability

The raw sequences described in this article were submitted and released to the National Genomics Data Center (NGDC, https://ngdc.cncb.ac.cn/gsa/search?searchTerm=CRA046203, accessed on 27 August 2026).

## Supplementary Materials

The following supporting information can be downloaded at https://www.mdpi.com/article/10.3390/plants15213496/s1, Table S1: Primers used for RT-qPCR validation.
