# Supplementary material for: Physiological and Transcriptomic Characterization of Salt-Stress Responses in the Pyrethrin-Producing Ornamental Plant Tanacetum coccineum
Source: Plants (Basel). 2026 Nov 9;15(21):3496. doi: 10.3390/plants15213496 (PMC13765814; doi:10.3390/plants15213496)
Supplement: Supplementary file 1 [file plants-15-03496-s001.zip › plants-4582433-supplementary.pdf]

Supplementary Material

Table S1. Primers used for qRT-PCR validation

ID	Primer name	Primer sequences（5' to 3'）
T. coccineum Realtime PCR	GLIP_RT_F	CGATGAACTTACTAGACTACACAA
T. coccineum Realtime PCR	GLIP_RT_R	AAGGACCACTACCGCAACAT
T. coccineum Realtime PCR	ADH_RT_F	GGTGAATGTGGACAATGCT
T. coccineum Realtime PCR	ADH_RT_R	CGGACCATGTGGAGCAACT
T. coccineum Realtime PCR	JMH_RT_F	TTTGTTGCGGATCTTTGGC
T. coccineum Realtime PCR	JMH_RT_R	GTCTTCCTGATCATGAGAATTGG
T. coccineum Realtime PCR	MT_RT_F	TTCATTCCTCAACCAGCCTACA
T. coccineum Realtime PCR	MT_RT_R	ACTATTTCGATAGAGCGTGATT
T. coccineum Realtime PCR	CHH_RT_F	CCACGTGTATGTAGGGAGCAATGC
T. coccineum Realtime PCR	CHH_RT_R	CAGGATCAGTTGAACATGCGAAAG
T. coccineum Realtime PCR	ALDH_RT_F	CATTCCGCTACTTTGCTGGTGC
T. coccineum Realtime PCR	ALDH_RT_R	TCCAAGGAATGATGTGTCCAACTAC
T. coccineum Realtime PCR	PYS_RT_F	CTACAAGGTGCCCCCAAAGA
T. coccineum Realtime PCR	PYS_RT_R	TGATGACGGCTCCAAGGTAGA
T. coccineum Realtime PCR	NCED3b_RT_F	TTTGCCAGAGGGATGGTTGG
T. coccineum Realtime PCR	NCED3b_RT_R	CAAGCTGGCCCCTCAAAAAC
T. coccineum Realtime PCR	ABA2b_RT_F	ACAATGCTGGGATTGGAGGG
T. coccineum Realtime PCR	ABA2b_RT_R	AGCAGCATGTTTCATCCCCA
T. coccineum Realtime PCR	AAO3b_RT_F	ACGAGGTGTCATTAAGGCCG
T. coccineum Realtime PCR	AAO3b_RT_R	AATGCCACCAACTTCCACCA
T. coccineum Realtime PCR	ZEPb_RT_F	AATCCACCAATCCCACCACC
T. coccineum Realtime PCR	ZEPb_RT_R	CACCGCGAAAAACGGAGAAA
T. coccineum Realtime PCR	GAPDH_RT_F	AAGGAGGAATCTGAAGGAAAGCTG
T. coccineum Realtime PCR	GAPDH_RT_R	GTTGTTGTTCAAAGCGATTCCAGC
